# Histotype-specific copy-number alterations in ovarian cancer

**DOI:** 10.1186/1755-8794-5-47

**Published:** 2012-10-18

**Authors:** Ruby YunJu Huang, Geng Bo Chen, Noriomi Matsumura, Hung-Cheng Lai, Seiichi Mori, Jingjing Li, Meng Kang Wong, Ikuo Konishi, Jean-Paul Thiery, Liang Goh

**Affiliations:** 1 Department of Obstetrics & Gynaecology, National University Hospital, 5 Lower Kent Ridge Road, Singapore, 119074, Singapore; 2 Cancer Science Institute, National University of Singapore, Centre for Life Sciences, #02-07 28 Medical Drive, Singapore, 117456, Singapore; 3 Cancer & Stem Cell Biology, Duke-National University of Singapore Graduate Medical School, 8 College road, Rm 6-32, Singapore, 169857, Singapore; 4 Department of Gynecology and Obstetrics, Kyoto University Graduate Medical School, Yoshida-Konoe-cho, Sakyo-ku, Kyoto, 606-8501, Japan; 5 National Defense Medical Center, Taiwan, 114 No.161, Sec. 6, Minquan E. Rd., Neihu Dist, Taipei City, 114, Taiwan; 6 Institute of Molecular and Cell Biology, 61 Biopolis Drive, Proteos, Singapore, 138673, Singapore; 7 Department of Medical Oncology, National Cancer Centre Singapore, 11 Hospital Drive, Singapore, 169610, Singapore; 8 Saw Swee Hock School of Public Health, Yong Loo Lin School of Medicine, National University of Singapore, MD3, 16 Medical Drive, Singapore, 117597, Singapore

**Keywords:** Ovarian cancer, Histological biomarkers, Genomics, Copy number driver genes, ERBB2

## Abstract

**Background:**

Epithelial ovarian cancer is characterized by multiple genomic alterations; most are passenger alterations which do not confer tumor growth. Like many cancers, it is a heterogeneous disease and can be broadly categorized into 4 main histotypes of clear cell, endometrioid, mucinous, and serous. To date, histotype-specific copy number alterations have been difficult to elucidate. The difficulty lies in having sufficient sample size in each histotype for statistical analyses.

**Methods:**

To dissect the heterogeneity of ovarian cancer and identify histotype-specific alterations, we used an *in silico* hypothesis-driven approach on multiple datasets of epithelial ovarian cancer.

**Results:**

In concordance with previous studies on global copy number alterations landscape, the study showed similar alterations. However, when the landscape was de-convoluted into histotypes, distinct alterations were observed. We report here significant histotype-specific copy number alterations in ovarian cancer and showed that there is genomic diversity amongst the histotypes. 76 cancer genes were found to be significantly altered with several as potential copy number drivers, including ERBB2 in mucinous, and TPM3 in endometrioid histotypes. ERBB2 was found to have preferential alterations, where it was amplified in mucinous (28.6%) but deleted in serous tumors (15.1%). Validation of ERBB2 expression showed significant correlation with microarray data (p=0.007). There also appeared to be reciprocal relationship between KRAS mutation and copy number alterations. In mucinous tumors where KRAS mutation is common, the gene was not significantly altered. However, KRAS was significantly amplified in serous tumors where mutations are rare in high grade tumors.

**Conclusions:**

The study demonstrates that the copy number landscape is specific to the histotypes and identification of these alterations can pave the way for targeted drug therapy specific to the histotypes.

## Background

Ovarian cancer is often dubbed a ‘silent’ killer because of its non-specific symptoms and late clinical onset which contribute to overall poor prognosis. There has been a steady increase in incidence over the last three decades with 204,000 new cases diagnosed each year globally [1]. It ranked fifth in mortality among cancers in women and has the highest case-fatality rate in gynecological cancers [2]. The 5-year survival rate for women with advanced disease remains at 29% with estimated 125,000 deaths annually [1,3].

About 90% ovarian cancers are epithelial ovarian cancer (EOC) [4], histologically subtyped as serous, mucinous, endometrioid, or clear cell. Further subtyping include the borderline cases, such as mucinous or serous borderline, often presented as stable diseases with more favorable outcome compared to the non-borderline subtypes. It is now recognized that epithelial ovarian cancer is a spectrum of diseases with varied genetic mutations among histotypes [5]. Genetically, mutations differ between the grades of the disease. Low-grade serous carcinoma have high frequency of KRAS and BRAF mutations but few p53 mutations while high grade serous carcinoma shows the inverse in frequency of these mutations [6,7]. Mucinous histotype has KRAS mutations [8] and endometrioid has PTEN mutations [9]. Despite the molecular heterogeneity, the treatment standard remains as taxane/platinum-based chemotherapy for all histotypes.

Genomic alterations such as copy number alterations (CNA) have been known to harbor drivers in carcinogenesis. Driver genes are genes that confer growth advantage on the cancer cells [10]. Several known copy number alteration drivers in cancers include receptor tyrosine kinases such as EGFR, FGFR, and ERBB2, which are targets for drugs therapy [11]. Successful incorporation of genomics alterations studies in cancer treatment has been evident in breast, leukemia, and lung cancers, where targeted therapies are part of the standard treatment protocols [12-14]. For example, Trastuzumab, a targeted therapy that can significantly reduce risk of disease recurrence and improve overall survival, is now standard of care for early-stage patients with Her2-positive breast cancer [14]. To date, targeted drug therapy has not been successfully incorporated in EOC.

One of the challenges in elucidating copy number alterations in EOC is the disproportional prevalence of histotype. Serous is the most prevalent (70-85%), followed by endometrioid (5-10%), clear cell (5-10%), and mucinous (~5%). The lower prevalent diseases tend to suffer from small sample size for statistical analyses to identify copy number alterations. To elucidate CNA in each histotype, we combined data from multiple studies with similar platforms to identify copy number alterations that are specific to the histotypes. The motivation is to identify high confidence histotype-specific alterations that may otherwise be obscured due to disproportionate prevalence of the histotypes.

## Results

Two major results are presented here; the histotype-specific copy number alterations for EOC and the identification of potential driver genes. Three datasets with corresponding gene expression and copy number profiling on similar platforms were used for this study. A hypothesis-driven approach using stringent false discovery rate (FDR) filtering was used to identify potential copy number driver genes (Methods). The genes were compared with other studies as well as cancer genes reported in literature. In addition, we also validated the expression of ERBB2, a driver gene, via quantitative real-time PCR (qPCR).

### Distinct copy number alterations in EOC histotypes

**Figure 1:**
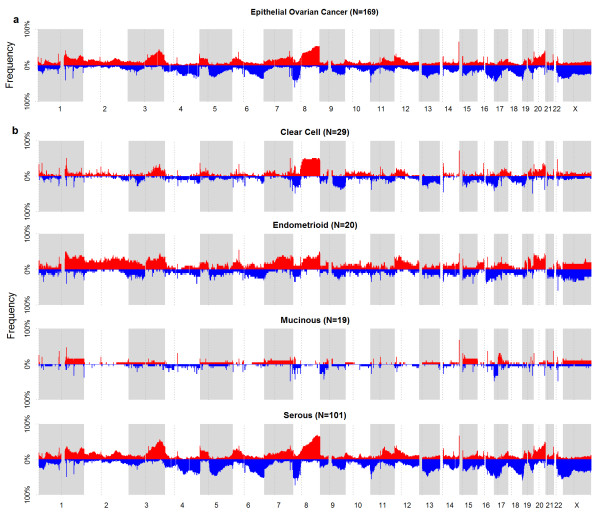
**Overview of copy number aberrations in EOC from 3 datasets from chromosomes 1**-**X.** Chromosomes are shown in alternating blocks of grey. The centromere for each chromosome is shown as a dotted line. (**a**) Frequency (%) of occurrence of amplification (red) and deletion (blue) from chromosome 1 to X in epithelial ovarian cancer from the merged 3 datasets. Major regions of alterations reported in other studies are similarly observed: e.g. chr 3, 8, 17, and 20. (**b**) Frequency (%) of occurrence in the 4 main histotypes of EOC. Threshold for frequency was set at LRR ≥ |0.2|. The frequency for serous tumors is similar to that in (**a**). However, frequency for the lower prevalent histotypes showed evident differences, indicating the molecular differences between the histotypes.

Figure 1a shows the global frequency of copy number aberrations for EOC across the genome for the merged dataset. In concordance with other reports, the four commonly reported chromosomes of 3q, 8, 17p, and 20q showed broad copy number alterations in EOC [15-17]. When this was de-convoluted into histotypes (Figure 1b, shown in 4 tracks for clear cell, endometrioid, mucinous, and serous), distinct differences were observed. It is evident that the general frequency for EOC was mirrored in serous tumors, not surprising since it has the largest sample size. In the four most commonly reported altered regions, 3q, 8, and 20q amplifications were observed in serous, clear cell, endometrioid but not mucinous tumors. Endometrioid and serous tend to harbor more copy number alterations, with more broad regions of alterations involving the p- or q-arm. The genomics landscape for clear cell and mucinous tumors appeared different from the other histotypes, with lesser broad regions of alterations and in lower frequency.

**Figure 2:**
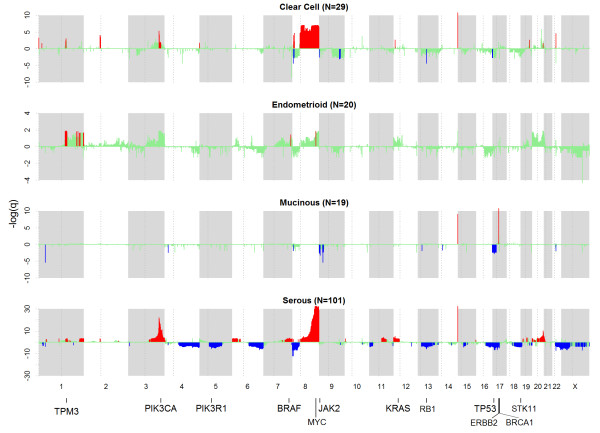
**Significance of copy number alterations (green) in clear cell, endometrioid, mucinous**, **and serous histotypes are shown in 4 horizontal tracks respectively.** GISTIC q-values (y-axis) are shown as –log_10._ Regions that passed the statistical selection criteria (see Methods) are shown as: amplification (red) and deletion (blue). Some known cancer and putative genes are indicated at the bottom of the plots. ERBB2 in chr17 is shown to be amplified in the mucinous track. Note scale difference in y-axis for the histotypes.

To assess the significance of copy number altered regions, we used a 2-pronged approach using merged and individual datasets (Additional file 1: Figure S1). Figure 2 shows significant copy number altered regions (see Methods) in the histotypes. Broad 3p amplification and 8p deletion were observed in serous tumors; 8q amplifications in clear cell and serous tumors; 17p deletions in mucinous and serous tumors; and chr20 amplifications in serous tumors. The nature of the alterations also differs, e.g. focal versus broad alterations. For example in 3q, serous tumors showed broader amplifications in the region of 3q13.31-29 while focal amplification was observed for clear cell tumors at 3q26.2-26.32. This is interesting as it has been reported that overlapping broad and focal aberrations can have distinct functional consequences [18]. In other chromosomes, alterations were specific to histotypes as well; such as 9p21 focal deletions in mucinous histotype, reportedly harboring homozygous deletions in EOC [19-22]. There are regions that displayed opposite trend in alterations between histotypes. One particular region, 8p23.1, showed amplification in clear cell but deletion in serous tumors. Another region which showed opposite trend in alteration was 17q12 which harbor the oncogene ERBB2; the gene was significantly amplified in mucinous but deleted in serous tumors. Excluding borderline cases, 28.6% (4/14) mucinous samples had amplifications and 15.2% (15/99) of serous tumors had deletions of ERBB2. 1/5 mucinous borderline also showed ERBB2 amplification. It should be of note that although ERBB2 was found significantly deleted in serous tumors, 5/99 (5.1%) of serous samples harbored the amplification, close to the 3% reported by TCGA for high grade serous [23]. No ERBB2 deletion was observed in the mucinous samples. As serous tumors have a comparatively larger sample size than the other histotypes, we would expect more significant regions for this histotype. Nevertheless, using stringent criteria, we were able to identify some significant CNA for the lower prevalent tumors.

**Figure 3:**
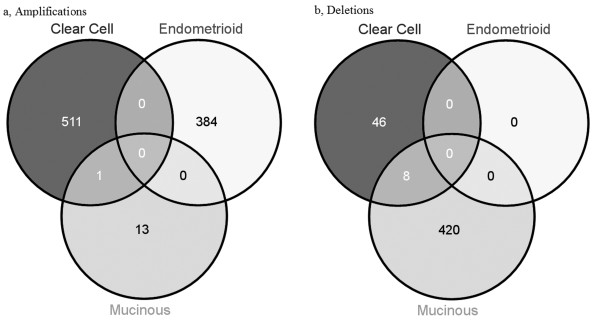
**Venn diagram of copy number altered genes between the lower prevalent histotypes.** Left: amplified genes; Right: deleted genes. Clear cell tumors had more amplified genes than deleted genes while mucinous tumors had more deleted genes than amplified genes.

To quantify genes that were altered in each histotype, we mapped genes to regions that were identified in each histotype. 6375 unique genes were found to be altered: 2682 amplifications and 3712 deletions (Additional file 2: Table S1). 91% of genes were amplified and 97.1% deleted in serous tumors, 19.1% amplified and 1.5% deleted in clear cell, 14.3% amplified in endometrioid, and 0.5% amplified and 11.5% deleted in mucinous. A total of 5360 genes were specific to each histotype (amplification=2014, deletion=3346), 5011 in serous tumors, 193 in endometrioid tumors, 79 in clear cell tumors, and 77 in mucinous tumors. Within each histotype, the type of alterations varied. Clear cell tumors had more amplified genes than deleted genes, while mucinous and serous tumors had more deleted genes than amplified genes. Only amplified genes were found in endometrioid tumors. Figure 3 shows the Venn diagram of overlapping genes in the lower prevalent histotypes. Due to the differences in sample size of each histotype, comparisons between overlapped genes were limited to genes found only in the non-serous tumors where the sample sizes are more comparable. A small number of overlapped amplified and deleted genes between clear cell and mucinous tumors were observed; none with endometrioid. This suggests that most of the CNA are specific to histotypes, adding to the mounting evidence of molecular differences between histotypes. Despite larger sample size in serous histotype, comparison of CNA found in the lower prevalent histotypes with serous is also of interest (Additional file 3: Figure S2). Clear cell tumors had the highest number of common altered genes with serous tumors (7.6%) while endometrioid tumors had the lowest number of common altered genes; 3.0% common altered genes with serous tumors only. There is also more overlap on amplifications than deletions.

### Consistency with other studies

Three studies have reported copy number changes in EOC [16,17,24] (Additional file 4: Table S2). These studies focused on global changes in EOC rather than histotype-specific alterations. Nevertheless, histotype-specific alterations from our study should overlap to some degree with these reported genes. We compiled a list of 551 significant genes reported in the 3 studies of which 545 genes were reported by either Haverty et al. or Gorringe et al. Eleven genes (2%) were commonly identified in at least 2 of the studies. In comparison, 39.2% (216/551) of genes were found in our study, indicating that our approach can identify copy number altered genes. We also compared our findings in serous tumors with TCGA high grade serous study (n=489) [23] where they reported 63 regions of gains and 50 regions of deletion. Based on overlapping of genes (if available) or genomic regions, 29/63 (46%) amplified and 27/50 (54%) deleted regions were also found in our study (n=101) (Additional file 5: Table S3).

### Copy number alterations in known cancer genes

300 cancer genes were previously reported [25], of which 76 cancer genes were found within the altered regions (Table 1). We found that copy number alterations of these cancer genes were specific to histotypes as well; e.g. TPM3 amplification in endometrioid tumors; JAK2 deletion in mucinous; RB1 deletion in both clear cell and serous tumors; TP53 and MAP2K4 deletion in mucinous and serous tumors. ERBB2, a gene implicated in breast and EOC showed significant focal amplification in mucinous tumors but deletions in serous tumors. Evaluation of ERBB2 expression between mucinous and serous tumors in the 3 datasets showed the trend of over expression of ERBB2 in mucinous compared to serous (Additional file 6: Table S4). The focal amplification of ERBB2 has been observed in various studies [26-28], supporting our findings.

**Table 1:** Summary of copy number alterations and potential driver cancer genes

**Region**	**AMP**				**DEL**				**Cancer genes**
	**C**	**E**	**M**	**S**	**C**	**E**	**M**	**S**	
1p36.11								D	MDS2
1q21.2-1q21.3		A							ARNT,**TPM3**
1q21.1-1q23.1		A		A					**BCL9**,MUC1,PRCC,NTRK1
1q42.13-1q44				A					**FH**
3q21.1-3q26.1				A					FOXL2,**GMPS**,MLF1
3q26.31-3q29				A					**PIK3CA**,ETV5,**EIF4A2**,BCL6,LPP,TFRC
4q21.22-4q31.3								D	RAP1GDS1,**TET2**,IL2,FBXW7
5q11.2-5q23.1								D	IL6ST,PIK3R1,APC
6p22.1-6p25.3				A					IRF4,DEK,HIST1H4I
6q22.2-6q27								D	ROS1,GOPC,STL,MYB,TNFAIP3,**FGFR1OP**,MLLT4
7q32.1-7q36.3				A					SMO,CREB3L2,KIAA1549,**BRAF**,EZH2
8p12-8p23.3								D	PCM1,WRN,WHSC1L1
8q11.21-8q24.3	A			A					HOOK3,**TCEA1**,CHCHD7,PLAG1,NCOA2,COX6C,EXT1,MYC,**RECQL4**
9p21.3-9p24.1							D		JAK2,MLLT3
11p15.4								D	CARS,NUP98,LMO1
11q13.3-11q21				A					**NUMA1**,PICALM,MAML2
12p11.21-12p13.33				A					KDM5A,CCND2,**ZNF384**,ETV6,KRAS
13q12.2-13q14.3								D	CDX2,FLT3,BRCA2,LHFP,LCP1
13q14.2					D			D	**RB1**
15q14-15q15.1								D	BUB1B
16q13-16q23.3								D	HERPUD1,CBFB,CDH1,MAF
17p11.2-17p13.2							D	D	USP6,TP53,PER1,GAS7,MAP2K4
17q11.1-17q21.31								D	**NF1**,SUZ12,TAF15,MLLT6,LASP1,RARA,BRCA1
17q12			A					D	**ERBB2**
18q21.32-18q22.2								D	MALT1,BCL2
19p13.3								D	FSTL3,STK11,TCF3,**SH3GL1**,MLLT1
20q11.21-20q13.33				A					ASXL1,GNAS,**SS18L1**
22q11.21-22q13.33								D	CLTCL1,MN1,CHEK2,EWSR1,NF2,MYH9,PDGFB,MKL1,MKL1,EP300
Xp11.3-Xp22.33								D	P2RY8,KDM6A
Xq25								D	ELF4

For simplicity, the altered regions are summarized into cytobands in the first column. Second (AMP) and third (DEL) columns indicate the amplification and deletion respectively for each histotype (C-clear cell, E-endometrioid, M-mucinous, S-serous). The amplification (A) and deletion (D) status for each histotype are indicated in each cytoband. The last column shows known cancer genes (from Sanger COSMIC database) that are within the altered regions. In total, 76 genes are listed in this table. Cancer genes which are potential drivers (i.e. significant correlation between gene expression and copy number alterations) are highlighted in bold.

### Identification of candidate driver genes in EOC histotypes

To identify candidate driver genes that might contribute to carcinogenesis of EOC, we looked for genes that showed association between copy number and gene expression (Methods, Additional file 1: Figure S1). Table 1 summarized the alterations and potential cancer driver genes (highlighted in bold) based on cytoband. Pathway analysis of potential driver genes in Table 1 showed top molecular functions of these genes to be involved with cell cycle, cellular development, growth and proliferation.

#### Candidate drivers in known cancer genes

**Figure 4:**
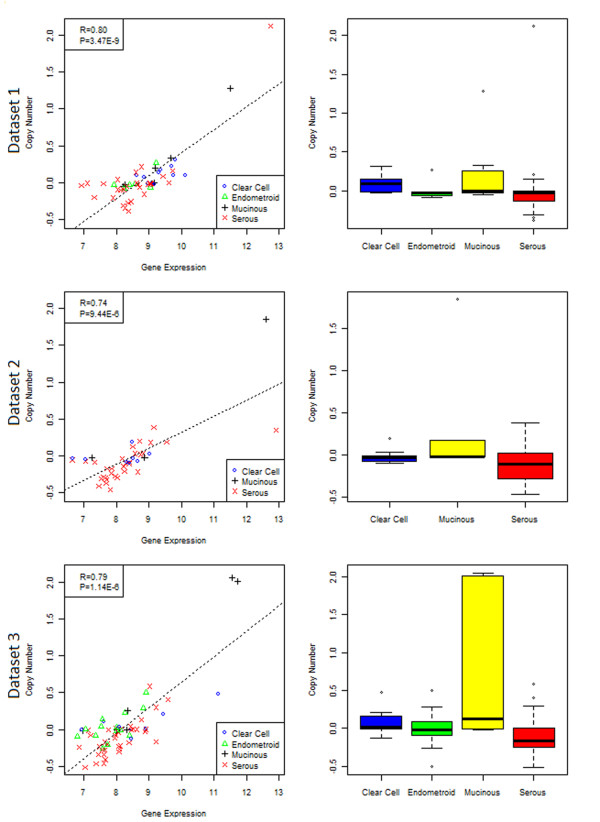
**Copy number and gene expression of ERBB2 for 3 datasets.** From top: dataset1, dataset2, dataset3. Each row shows (left) the correlation between copy number and gene expression, and (right) boxplot of copy number across histotypes. Significant correlations were observed between gene expression and copy number alterations, suggesting a potential copy number driver mechanism.

Among 76 identified cancer genes listed in Table 1, several genes were potential drivers (highlighted in bold) in EOC in the association analysis. These include: FH, GMPS, PIK3CA, EIF4A2, ZNF384, and SS18L1 (amplifications in serous tumors); TET2, FGFR10P, NF1, ERBB2, and SH3GL1 (deletions in serous tumors) and ERBB2 (amplifications in mucinous tumors). Figure 4 shows the association between copy number and gene expression of ERBB2. The correlation was significant for all 3 datasets (Dataset1: R=0.80, p=3.47E-09, Dataset2: R=0.74, p=9.44E-6, Dataset3: R=0.79, p=1.14E-6, meta-p=3.90E-17), suggesting a driver mechanism. Amplification was observed in mucinous and deletion in serous tumors. Another interesting observation was MYC, TP53, KRAS, and BRCA1, genes reportedly to be commonly mutated in cancers but did not show significant association between copy number and gene expression. Similarly, two other genes reported to be mutated in EOC (PIK3R1 and STK11) also did not show potential driver mechanism.

### Validation of ERBB2 expression

**Figure 5:**
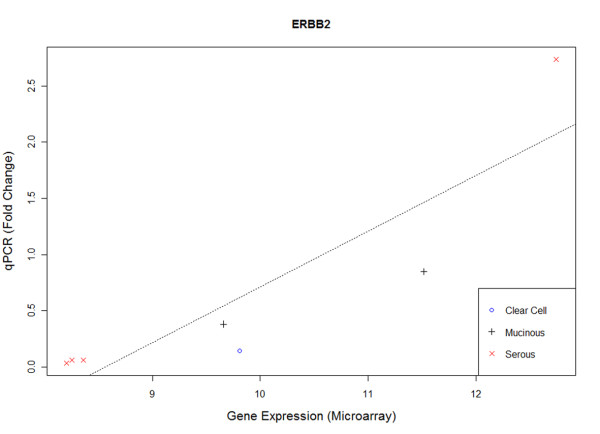
**Scatter plot of ERBB2 gene expression and qPCR of 7 samples identified to be copy number altered in Dataset1.** 4 ERBB2 amplified samples (serous, mucinous, mucinous borderline, and clear cell) had higher expression than the 3 ERBB2 deleted serous samples. Significant correlation was observed between gene expression and qPCR (p=0.007).

It is not within the scope of this study to validate the candidate copy number driver genes. As ERBB2 has potential for targeted therapy, we validated the expressions of ERBB2 via qPCR of 7 samples in Dataset1 that were found to be amplified or deleted. It should be noted that no more samples were available in Dataset2 for validation. Figure 5 shows the scatter plot between gene expression from microarray and qPCR (measured as fold-change). Significant correlation was observed (p=0.007) between microarray and qPCR. Four ERBB2 amplified samples (1 serous, 1 mucinous, 1 mucinous borderline, and 1 clear cell) were expressed correspondingly higher than the 3 serous samples with deleted ERBB2 (p=0.06, Wilcoxon). All these data support ERBB2 as a copy number driver gene in EOC.

## Discussion

The study reveals genomics diversity in EOC. It is conceivable that some of these alterations are involved in the tumorigenesis of EOC but the pathogenesis is likely regulated by aberrations of histotype specific alterations. By stratifying based on histotypes, we were able to identify alterations in the lower prevalent clear cell, endometrioid, and mucinous samples. An example is ERBB2. Several groups have investigated alterations of ERBB2 in EOC with mixed results [29,30]. However, when stratified into histotypes, the high prevalence of ERBB2 amplification in mucinous is clearly evident in our results and other studies [26-28]. In addition, our results support ERBB2 as a potential copy number driver gene in EOC. The differences of ERBB2 copy number alterations amongst the histotypes could be due to the origin of histotypes in EOC. Our study demonstrates the importance of histotype-specific analyses where the differing copy number landscape amongst the histotypes adds to the mounting evidence that EOC should not be treated as one disease.

76 cancer genes listed in Table 1 were found to be copy number altered in EOC. They include ERBB2, TPM3, BRCA1, BRAF, KRAS, and PIK3CA; some of which are potential copy number drivers e.g. PIK3CA and BRAF in serous histotypes. Another interesting observation was KRAS, a gene reported to be mutated in mucinous tumors. In our study, KRAS was not found significantly altered in mucinous tumors where mutations are common. However, KRAS was significantly amplified in serous tumors where mutations are rare in high grade tumors. The reciprocal relationship between KRAS mutation and copy number alterations is also observed in gastric cancer [11]. The 8q24.21 region harboring MYC, on the other hand, was altered in most histotypes other than mucinous. TCGA study also indicates MYC is highly amplified in high grade serous tumors. This suggests that MYC inhibitors may be applicable for these histotypes. For cancer genes which have been reported to have somatic or germline mutations in EOC, 3 were found to harbor copy deletions in serous histotype: PIK3R1, BRCA1, and STK11. For BRCA1, our finding was concordant with previous report that BRCA1 locus could be lost via either deletion or epigenetic silencing other than mutation in sporadic EOC [31].

A number of the candidate drivers in Table 1 are also implicated in translocation aberrations, e.g. TPM3, BCL9, GMPS, ZNF384 and SS18L1. It’s interesting that these genes were amplified in endometrioid and/or serous tumors. TPM3 and BCL9 reside in 1q21, a frequent site for chromosomal rearrangements. TPM3 was specifically amplified in endometrioid tumors and the gene has been shown to constitute a fusion gene with NTRK1 which belongs to the group of TRK oncogenes reported for papillary thyroid carcinoma [32]. Interestingly, NTRK1 is also significantly amplified only in endometrioid tumors and further investigation is required to ascertain if this is due to gene fusion. BCL9 is a novel oncogene in Wnt signaling pathway, playing a critical role in epithelial-mesenchymal transition in colon epithelium and adenocarcinomas [33,34]. Translocation of BCL9 has been reported with 14q32 [35] and the gene was amplified in both endometrioid and serous tumors. Translocations for GMPS, ZNF384, and SS18L1 were also found in leukemia and synovial sarcomas [36-40] and all were amplified in serous tumors.

There are several drugs targeting the genes, e.g. for ERBB2, inhibitors include Trastuzumab, Lapatinib, and Pertuzumab. Lately, a clinical trial on combination of Pertuzumab, Trastuzumb and Docetaxel improved outcome of patients with HER2 positive metastatic breast cancer [41]. BRAF mutations are more common in low grade serous while BRAF amplification is more common in high grade serous. Our data showed that it is a potential copy number driver and hence may be targetable by BRAF-inhibitors in serous tumors. Most BRAF inhibitors target various mutations and its efficacy on amplified BRAF is not yet well understood. A study has shown that BRAF amplified colorectal cancer cells acquired resistance to the MEK1/2 inhibitors selumetinib [42]. PIK3CA is significantly amplified in serous histotypes and could be a potential target for PI3K inhibitors. In a study of PI3K inhibitor on breast and gynecologic malignancies harboring PIK3CA mutations, patients with the mutations treated with the inhibitor showed higher response rate than patients without the mutations [43].

In combining the 3 datasets, there was concern with regards to the genetic diversity amongst the Chinese Japanese, and Caucasian samples. The Hapmap [44] and Human Genome Diversity projects [45] have showed that these ethnic groups are different, though Chinese (CHB) and Japanese (JPT) tend to have high similarity in population structure. As genetic differences can be evaluated via principal component analysis (PCA) [46], we used PCA to assess the copy number data of the 3 cohorts. No distinct clustering between the groups (Additional file 7: Figure S3) was observed, suggesting that in this particular copy number landscape, the genetic effect is not evident and therefore has minimal effect in the analyses. We also used ANOVA test to assess ~200 housekeeping genes between the 3 datasets; none of the genes showed significance (Additional file 8: Table S5). Note that ERBB2 also did not show any significance. Nevertheless, genetic differences were taken into consideration in the preprocessing protocol. Individual dataset was normalized with respect to the relevant ethnic group from Hapmap data, i.e. Dataset1 with JPT, and Dataset2 with CHB. Ethnic-specific common structural polymorphism was also filtered out (see Data analysis) to ensure the copy number alterations identified in this study are de novo alterations in tumors.

We recognize that the regions identified could still be limited by the individual sample size of the histotypes. The larger number of copy number altered genes in serous tumors could be attributed to the larger sample size in this collection. We performed sub-sampling analyses to ascertain the effects and in addition, to ensure robustness of results, we used stringent criteria to filter the regions as well as criteria to consider CNA genes if it were supported by at least 2 dataset (Additional file 1: Figure S1). The flip side of this filtering was that true regions of alterations could be filtered out (as shown in the green area in Figure 1), leading to probably more false negatives. Nevertheless, we observed that despite the filtering and limited sample size of some histotypes, significant regions were still observed in the less prevalent histotypes; e.g. the 1p36.33, 2p11.1, 19q13.31, and 20q13.33 amplification and 9q32 deletion in clear cell tumors (n=29); 1q21.2-3 amplification in endometrioid tumors (n=20); 17q12 amplification and p24.1 deletion in mucinous tumors (n=19). Note that endometrioid tumors were not available in Dataset 2 although the total number of tumors was comparable with clear cell and mucinous. The concordance criteria of agreement on 2 datasets in the analytical workflow would thus bias the identification of regions for this histotype. Despite this, significant alterations were still observed for endometroid (e.g. TPM3) and given the stringent criteria; these are likely high confidence alterations. It should be noted that the samples were stratified according to the 4 main histotypes, including some borderline cases. Although borderline cases are presented clinically as a different subtype, they were included to simplify the stratification of histotypes and analyses. The significance of this approach can be seen in ERBB2, where both mucinous and mucinous borderline cases harbor amplification and corresponding up regulation of expression as well. This was similarly observed in other studies [26-28]. To assess if copy number alterations differ between borderline and non-borderline tumors and would thus cause bias in our analyses, we evaluated PCA of these samples (Additional file 9: Figure S4). No distinct clustering was observed between the borderline and non-borderline groups.

## Conclusions

In summary, our study showed genomic diversity in EOC and highlighted distinct copy number alterations in histotypes that may have potential for drug targeted therapy. ERBB2 is significantly amplified in mucinous tumors and is a candidate copy number driver gene. By merging multiple datasets of similar platform, we demonstrated that CNA in the lower prevalent histotypes could be elucidated, even with limited sample size.

## Methods

### Dataset1

56 archived frozen tumor samples from the Department of Gynecology & Obstetrics, Kyoto University Graduate School of Medicine, Japan were profiled on microarray. It contained 12 clear cell carcinoma, 6 endometrioid adenocarcinoma, 2 mucinous adenocarcinoma, 5 mucinous-borderline tumors, 26 serous adenocarcinoma, and 5 serous-borderline tumors.

### Dataset2

46 archived frozen tumor samples collected from Department of Obstetrics and Gynecology, Tri-Service General Hospital, Taiwan, containing 9 clear cell, 6 mucinous, and 31 serous.

### Dataset3

GSE19539 consisting of 8 clear cell, 14 endometrioid, 6 mucinous, and 39 serous [15]. Blood normal available in the dataset was used for normalization in concordance with the paper.

### Human ovarian carcinoma samples

Two collections of archived flash frozen ovarian carcinoma samples were obtained from the Department of Gynecology & Obstetrics, Kyoto University Graduate School of Medicine, Japan (Dataset1, N= 56) and Department of Obstetrics and Gynecology, Tri-Service General Hospital, Taiwan (Dataset2, N=46). All samples were collected with the donor’s written informed consent. Ethical clearance has been approved by the Institutional Review Board for both institutes. All samples were reviewed by at least one pathologist from the respective institutes on histopathological typing and purity of samples. Tumor genomic DNAs were extracted by using phenol-chloroform extraction method. Tumor RNAs were extracted by using Qiazol followed by column clean-up using miRNeasy kit (Qiagen).

### Copy number profiling

Affymetrix Genome-Wide Human SNP Arrays 6.0 (Affymetrix, Santa Clara, California) were used for copy number analysis according to the cytogenetics protocol from the manufacturer. Data was pre-processed and normalized with Hapmap JPT or CHB for Japan and Taiwan samples respectively using the Affymetric Genotyping Console. Copy number segments were obtained from the circular binary segmentation (CBS) algorithm [47] implemented in R package DNAcopy using default settings.

### Gene expression profiling

Affymetrix GeneChip Human Gene 1.0ST Array was used for gene expression analysis according to the protocols from the manufacturer. Data was pre-processed and RMA normalized [48] using Affymetrix Gene Expression Console. Expressions for genes were mean-aggregated for each gene based on Affymetrix probes annotation. Note: 3 mucinous samples in Dataset2 and 2 serous samples in Dataset3 do not have corresponding gene expression data.

### Data repository

The gene expression and copy number datasets are MIAME compliant and have been submitted to National Centre for Biotechnology Information’s (NCBI) Gene Expression Omnibus (GEO) website, series accession number GSE30311.

### Quantitative real time PCR

Total and miRNA was isolated from the ovarian carcinoma tissues using the miRNeasy kit (QIAGEN), of which 500ng were used to generate cDNA using the RT^2^ first strand kit (QIAGEN). For the qPCR run, 200ng of first strand cDNA was used per gene analysis. To determine the expression profile, ERBB2 transcript expression levels were normalized against the averaged expression levels of 5 housekeeping genes (ACTB, B2M, GADPH, HPRT and RPL13A).

### Delta-C_t_ (ΔC_t_) and fold-change determination

C_t_ was determined using the SDS software (version 2.3, Applied Biosystems). Briefly, C_t_ values were determined by setting the baseline between cycle 2 of the run (total run: 40 cycles) and 2 cycles before the start of the first log-phase amplification. The threshold was set by positioning the limit to the lower third of the earliest amplification. ΔC_t_ was calculated by the formulae:

(1) $$\Delta C_{t}=C_{t}\left(\text{GOI}\right)–C_{t}\left(\text{HKG}\right)$$

whereby: C_t_ (GOI): C_t_ value of the respective gene of interest (GOI), C_t_ (HKG): average C_t_ values of the 5 housekeeping genes (HKG) used in the assay, Fold-change of the transcript is determined by the following formula:

(2) $$\text{Fold}-\text{change}=2^{\left(-\Delta \text{Ct}\right)}$$

### Data analysis

To identify significant copy number altered regions, we used a 2-pronged workflow employing the GISTIC algorithm [18]. GISTIC identifies copy number alterations based on the frequency as well as the log relative ratio (LRR) signals to compute the q value (false discovery rate). Default settings were used in the GISTIC analysis, and amplification and deletion thresholds were set at 0.2 and −0.2 respectively. Additional file 1: Figure S1 shows the 2-pronged workflow involving merged and individual copy number datasets to identify copy number alterations. Alterations were considered significant if it passed the following filtering criteria: (i) q < 0.25 (individual dataset), (ii) q < 0.05 (merged dataset), and (iii) concordance in 2 or more datasets. The significant regions were than mapped to genes (hg18 Refseq) by averaging the segments within each gene. ANOVA test was used to identify histotype-specific alterations. The analyses resulted in a list of significant gain and loss genes for each histotype, summarized in Figure 2 and Table 1 (known cancer genes).

To identify potential driver genes, non-parametric Spearman correlation was used to assess association between gene expression and copy number alterations of individual gene for each dataset (Additional file 1: Figure S1). Fisher’s combined probability test (meta-p) [49] was then used to combine the correlation statistics from each dataset to identify potential driver genes. This hypothesis driven association approach has been used to identify potential cancer driver genes [50,51]. Potential driver genes of known cancer genes are listed in Table 1 (in bold).

PCA plots were generated using Partek Genomics Suite (Partek, Missouri, USA). Pathway analyses were performed using Ingenuity Pathway Analysis software (Ingenuity, California, USA). The frequency plot for copy number altered regions in Figure 1 was generated using the threshold of LRR ≥ |0.2|. All statistical analyses and plots were done using the R programming package (http://www.r-project.org).

### Sub-sampling analyses to ascertain effects of sample size

To assess the effects of disparate sample size of the histotypes in the merged copy number data, multiple sub-sampling (with replacement) on the merged serous tumors was performed to ascertain the false positive and negative. The results showed that ≥97% of genes identified in sample size of 20–30 were also found in sample size of 101. However, 43-57% of genes found in the larger sample size were not identified in the smaller sample size datasets. In view of this, we have mainly confined comparison of genes found in the non-serous tumors.

## Abbreviations

EOC: Epithelial ovarian cancer; PCR: Polymerase chain reaction; qPCR: Quantitative real-time polymerase chain reaction; FDR: False discovery rate; TCGA: The cancer genome atlas; PCA: Principal component analysis; CHB: Hapmap Chinese; JPT: Hapmap Japanese; CBS: Circular binary segmentation; NCBI: National Centre for Biotechnology Information; GEO: Gene expression omnibus; GOI: Gene of interest; HKG: Housekeeping genes; GISTIC: Genomic identification of significant targets in cancer; ANOVA: Analysis of variance.

## Competing interests

The authors declare no competing interests.

## Authors’ contributions

RYJH, JPT, and LG drafted the manuscript. GBC and LG performed the analyses. NM, HCL, IK collected the tissues. RYJH, SM, MKW extracted the RNA and DNA for profiling. RYJH and MKW carried out the PCR. LG conceived of the study. All authors read and approved the final manuscript.

## Pre-publication history

The pre-publication history for this paper can be accessed here:

http://www.biomedcentral.com/1755-8794/5/47/prepub

## Supplementary Material

Additional file 1: Figure S1 Data Analysis Workflow. Two pronged approach for individual and merged datasets through selective threshold of GISTIC q-value and concordance in copy number analysis. As some histotypes have lower prevalence, filtering thresholds for individual and merged dataset were set at q<0.25 and q<0.05 respectively to overcome differences in sample size. In addition, any genomic alterations are supported by at least 2 datasets (i.e. concordance criteria). Specifically, the filtering criteria for histotype-specific regions were: (i) q < 0.25 (individual dataset), (ii) q < 0.05 (merged dataset), and (iii) concordance in 2 or more datasets. This resulted in a list of significant gains and loss regions. To identify copy number driver genes that are specific to histotype, copy number segments were mapped to genes and ANOVA was used to identify the differentially altered genes. This resulted in a list of histotype-specific altered genes. Spearman correlation between gene expression and copy number was then used to assess potential driver genes in each individual dataset. Click here for file

Additional file 2: Table S1 Summary of overlapped amplified and deleted genes between histotypes. Click here for file

Additional file 3: Figure S2 Venn diagram of copy number altered genes between the 4 histotypes. Left: amplified genes; Right: deleted genes. Clear cell tumors had the highest number of common altered genes with serous tumors while endometrioid tumors had the lowest number of common altered genes. Click here for file

Additional file 4: Table S2 Summary of datasets used for comparison of commonly altered genes. Click here for file

Additional file 5: Table S3 Summary of comparison with TCGA. Click here for file

Additional file 6: Table S4 Comparison of ERBB2 expression between mucinous and serous tumors. Click here for file

Additional file 7: Figure S3 Principal component analysis of copy number altered gene from the merged datasets. The plot shows that there is minimal copy number alterations difference between the 3 datasets. Click here for file

Additional file 8: Table S5 Summary of anova results on the 3 datasets. Click here for file

Additional file 9: Figure S4 Principal component analysis of copy number altered genes from the merged datasets showing borderline and non-borderline tumors. Borderline tumors were available only in serous and mucinous histotypes. No distinct clustering was observed between borderline and non-borderline tumors for (a) mucinous and (b) serous. Click here for file
