# Supplementary figures and images for: Histotype-specific copy-number alterations in ovarian cancer
Source: BMC Med Genomics. 2012 Oct 18;5:47. doi: 10.1186/1755-8794-5-47 (PMC3567940; doi:10.1186/1755-8794-5-47)

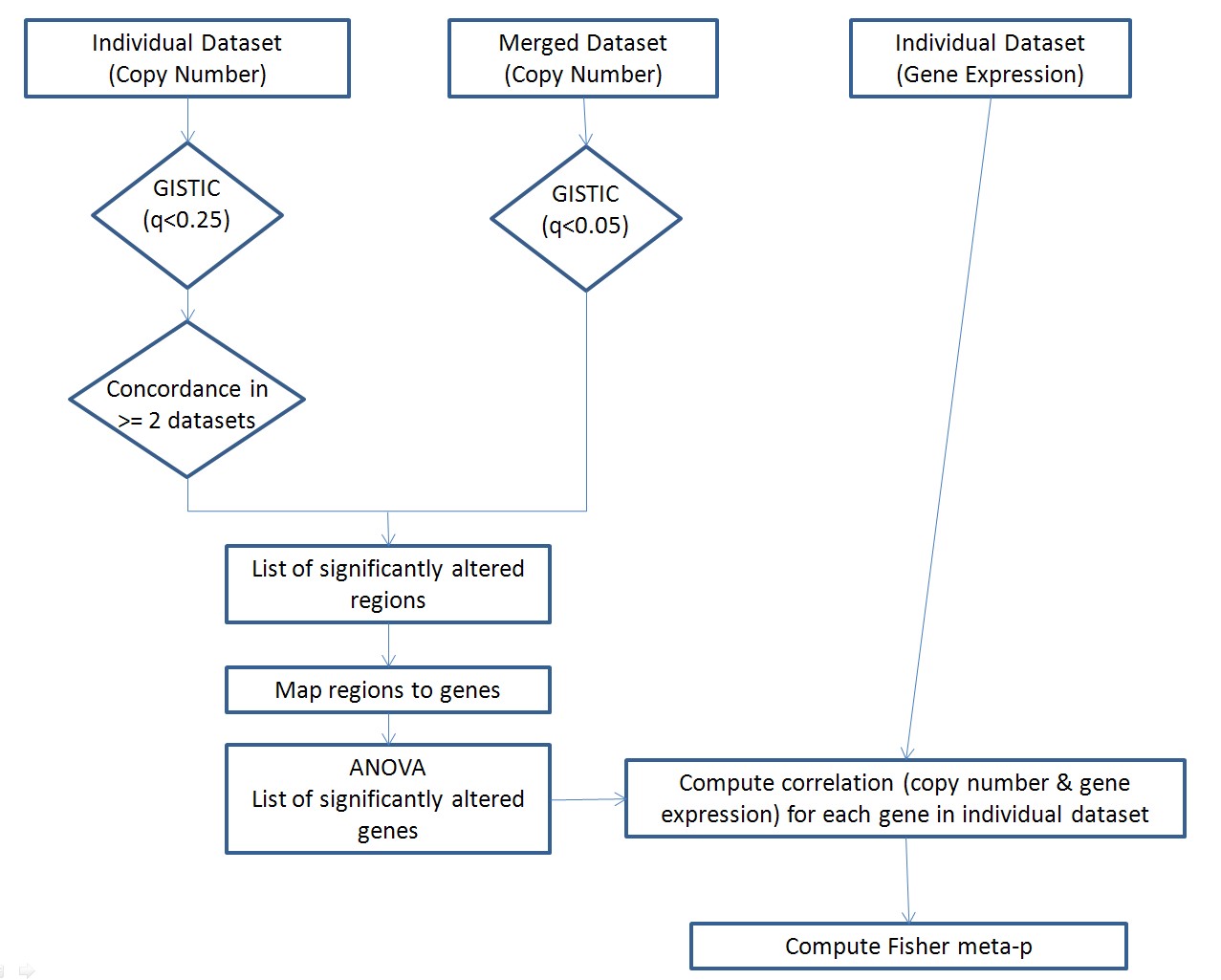

Supplement: Additional file 1: Figure S1 — Data Analysis Workflow. Two pronged approach for individual and merged datasets through selective threshold of GISTIC q-value and concordance in copy number analysis. As some histotypes have lower prevalence, filtering thresholds for individual and merged dataset were set at q<0.25 and q<0.05 respectively to overcome differences in sample size. In addition, any genomic alterations are supported by at least 2 datasets (i.e. concordance criteria). Specifically, the filtering criteria for histotype-specific regions were: (i) q < 0.25 (individual dataset), (ii) q < 0.05 (merged dataset), and (iii) concordance in 2 or more datasets. This resulted in a list of significant gains and loss regions. To identify copy number driver genes that are specific to histotype, copy number segments were mapped to genes and ANOVA was used to identify the differentially altered genes. This resulted in a list of histotype-specific altered genes. Spearman correlation between gene expression and copy number was then used to assess potential driver genes in each individual dataset. [file 1755-8794-5-47-S1.jpeg]

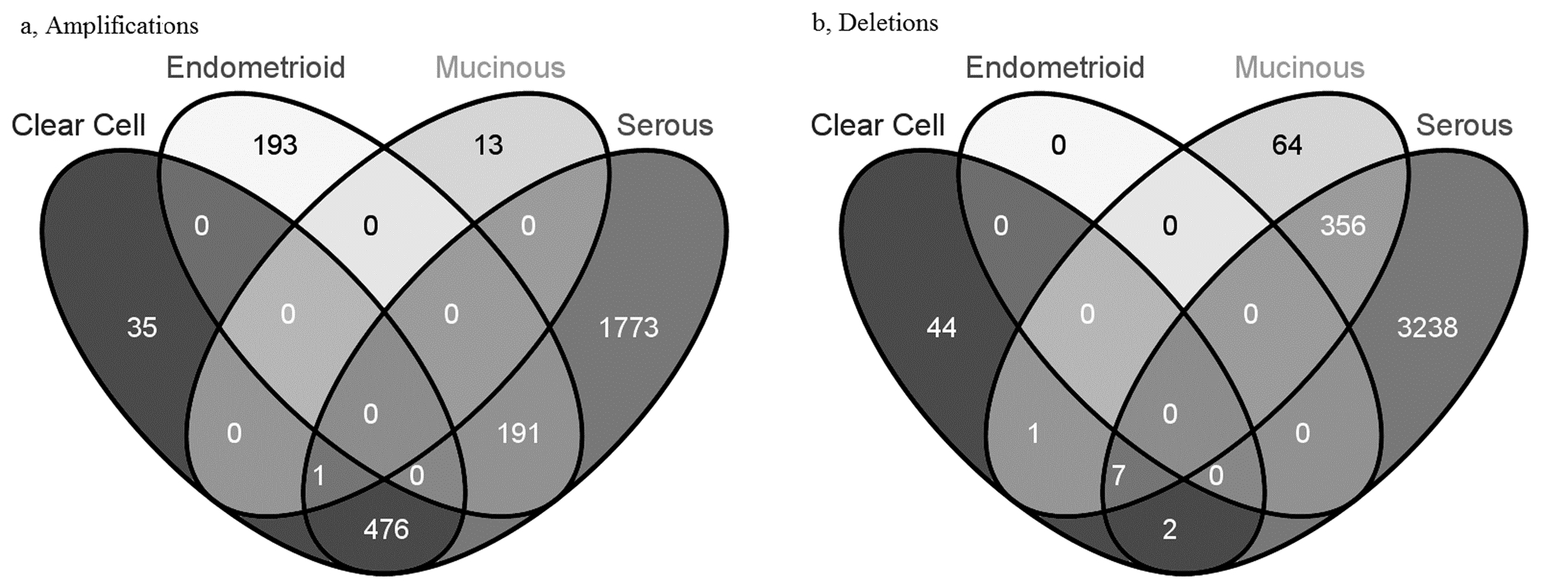

Supplement: Additional file 3: Figure S2 — Venn diagram of copy number altered genes between the 4 histotypes. Left: amplified genes; Right: deleted genes. Clear cell tumors had the highest number of common altered genes with serous tumors while endometrioid tumors had the lowest number of common altered genes. [file 1755-8794-5-47-S3.jpeg]

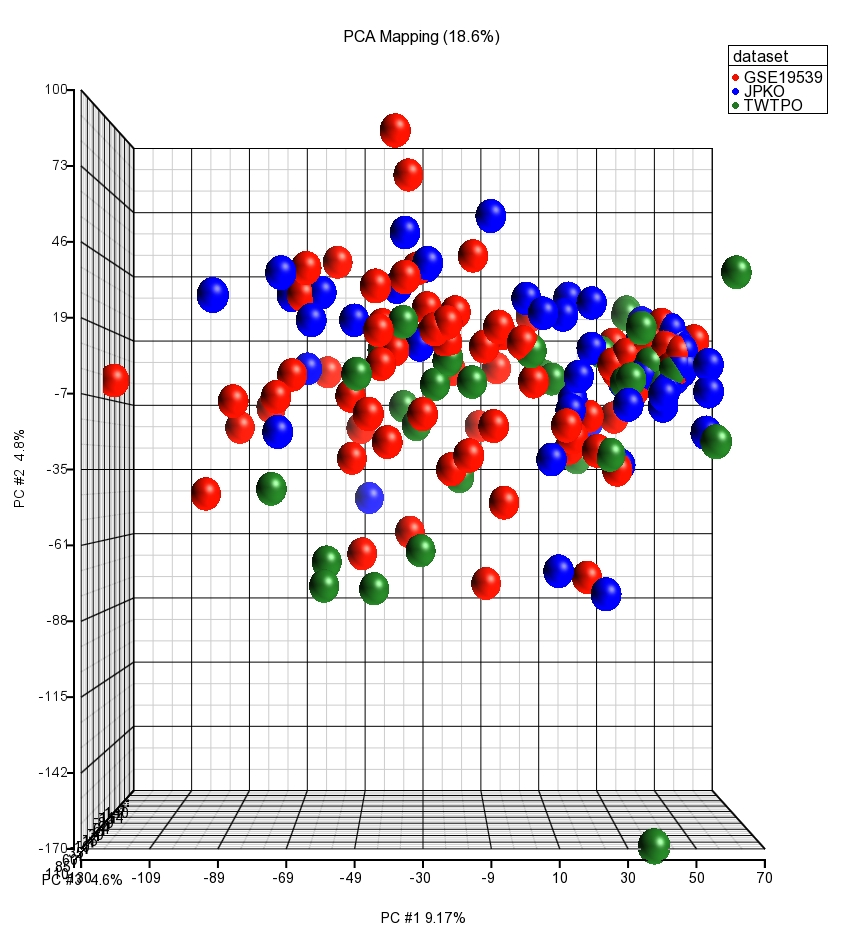

Supplement: Additional file 7: Figure S3 — Principal component analysis of copy number altered gene from the merged datasets. The plot shows that there is minimal copy number alterations difference between the 3 datasets. [file 1755-8794-5-47-S7.jpeg]

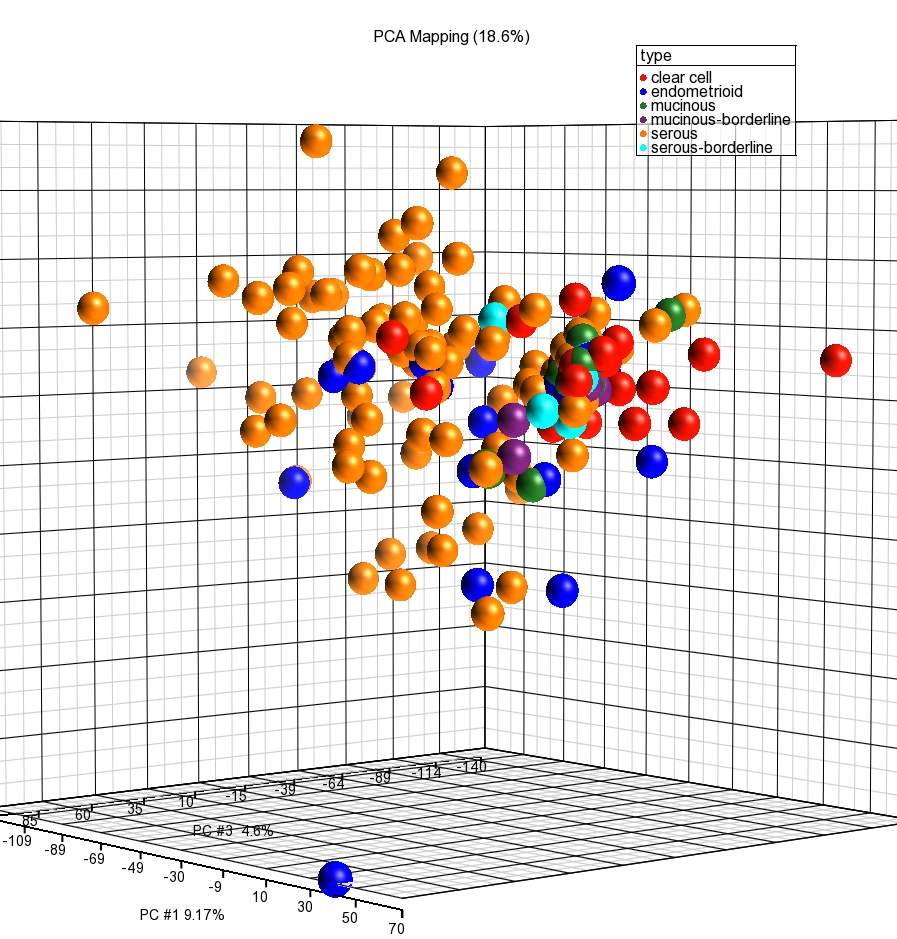

Supplement: Additional file 9: Figure S4 — Principal component analysis of copy number altered genes from the merged datasets showing borderline and non-borderline tumors. Borderline tumors were available only in serous and mucinous histotypes. No distinct clustering was observed between borderline and non-borderline tumors for (a) mucinous and (b) serous. [file 1755-8794-5-47-S9.jpeg]
