# Supplementary material for: Histotype-specific copy-number alterations in ovarian cancer
Source: BMC Med Genomics. 2012 Oct 18;5:47. doi: 10.1186/1755-8794-5-47 (PMC3567940; doi:10.1186/1755-8794-5-47)
Supplement: Additional file 2: Table S1 — Summary of overlapped amplified and deleted genes between histotypes. [file 1755-8794-5-47-S2.doc]

Table S1: Summary of overlapped amplified and deleted genes between histotypes.

|  | **Amplification (Unique genes=2682)** | | | | **Deletion (Unique genes=3712)** | | | |
| --- | --- | --- | --- | --- | --- | --- | --- | --- |
|  | Clear Cell (%) | Endometrioid (%) | Mucinous (%) | Serous (%) | Clear Cell (%) | Endometrioid (%) | Mucinous (%) | Serous (%) |
| Clear Cell | 512 (19.1) | 0 (0) | 1 (0) | 477 (17.8) | 54 (1.5) | 0 (0) | 8 (0.2) | 9 (0.2) |
| Endometrioid |  | 384 (14.3) | 0 (0) | 191 (7.1) |  | 0 (0) | 0 (0) | 0 (o) |
| Mucinous |  |  | 14 (0.5) | 1 (0) |  |  | 428 (11.5) | 363 (9.8) |
| Serous |  |  |  | 2441 (91) |  |  |  | 3603 (97.1) |
