# Supplementary material for: Histotype-specific copy-number alterations in ovarian cancer
Source: BMC Med Genomics. 2012 Oct 18;5:47. doi: 10.1186/1755-8794-5-47 (PMC3567940; doi:10.1186/1755-8794-5-47)
Supplement: Additional file 4: Table S2 — Summary of datasets used for comparison of commonly altered genes. [file 1755-8794-5-47-S4.doc]

Table S2: Summary of datasets used for comparison of commonly altered genes

| **Publication** | **Platform** | **Samples Info** | **# of genes reported** |
| --- | --- | --- | --- |
| Haverty, P. M., L. S. Hon, et al. (2009) | Affymetrix 500K SNP array | 57 (36 serous, 9 mullerian mixed, 4 unspecified, 3 clear cell, 2 endometrioid, 2 mucinous, 1 granulosa cell tumor) | 475 |
| Gorringe, K. L., S. Jacobs, et al. (2007) | Affymetrix 500K SNP array | 31 (11 serous,9 mucinous and 11 endometrioid) | 74 |
| Gorringe, K. L., M. Ramakrishna, et al. (2009) | Affymetrix 500K & SNP6.0 | 125 (63 serous, 18 mucinous, 28 endometrioid, 11 clear cell, and 5 other) | 12 |
